# Genetic variability of the bollworm, Helicoverpa armigera, occurring on different host plants

**DOI:** 10.1673/2006_06_26.1

**Published:** 2006-09-28

**Authors:** S. Subramanian, S. Mohankumar

**Affiliations:** Department of Plant Molecular Biology and Biotechnology, Centre for Plant Molecular Biology, Tamil Nadu Agricultural University, Coimbatore – 641 003, India

**Keywords:** insect diversity, Simple Sequence Repeat markers, tomato, chili, cotton, chickpea, blackgram, redgram, SSR simple sequence repeat

## Abstract

The bollworm, Helicoverpa armigera Hübner (Lepidoptera: Noctuidae) is a polyphagous pest of worldwide occurrence inflicting annual crop damage in India worth US$ 1billion. In India this insect occurs as a major pest in many economically important crops, including cotton, pigeonpea, chickpea, tomato, okra, and blackgram. Understanding the genetic variation among the H. armigera populations occurring on host plants has become essential to understand the variation in their susceptibility to different insecticides, including Bacillus thuringiensis. This preliminary study uses 10 microsatellite simple sequence repeat (SSR) markers, to provide insight into the genetic variability of H. armigera populations from six different host plants. Nine of the SSR primers indicated high variability across the different host associated populations with polymorphism ranging from 75 to 100 per cent. Using the un-weighted pair-group method analysis, H. armigera collected and reared from cotton stood out as unique in one cluster while the insects collected and reared on all other hosts grouped separately.

## Introduction

Helicoverpa armigera Hübner (Lepidoptera: Noctuidae) is a very destructive polyphagous pest occurring on cotton, tomato, bhendi, chickpea, pigeonpea, chilli, maize, sorghum and many other crops, inflicting substantial crop losses every year (Reed and Pawar 1982; Manjunath et al.1989; Sharma 2001). The ability of insect species to thrive on diverse host plants is an adaptive advantage for their better survival in the ecosystem. H. armigera is also characterized by its high mobility and fecundity and it has shown great capacity to develop resistance to synthetic insecticides used in its management (Armes et al. 1996; Kranthi 1997; Ramasubramaniam and Regupathy 2004). However in polyphagous insects, colonization of a new host may induce the selection of adaptive characters and genetic differentiation in population (Rice 1987; Diehl and Bush 1989).

In nature, polyphagous pests tend to be mono or oligophagic at the micro ecological level and their populations could be made up of individuals that are predominantly monophagous (Karowe 1989). Hence polyphagy at the species level, as has been demonstrated in H. armigera, does not necessarily imply polyphagy at the individual level (Cunningham et al. 1999). The selective use among diverse resources may lead to the evolution of ecological specialization and adaptation (Berenbaum 1996; Kawecki 1997). The versatility of this species may be due to the presence of a strong genetic variability governing the behavior of H. armigera (Zhou et al. 2000;Scott et al. 2003) making it a serious pest on several crops. In this regard a better understanding of the genetic differences of polyphagous pest like H. armigera can be very useful to understand the structure and population dynamics, their behavior and response to various selection pressures.

Ravi et al. (2005) found that the relative abundance of H. armigera in redgram and chickpea was much higher than in cotton and other host crops in a South Indian cotton ecosystem. The genetic variation among geographic populations of H. armigera collected from the South Indian cotton ecosystem was analyzed using RAPD markers and 12 populations could be classified into two distinct groups (Fakrudin et al. 2004).

In the present study the genetic variability of H. armigera occurring on six different host plants were analyzed using simple sequence repeat (SSR) markers. The characteristics of SSR markers such as coverage of multiple loci, co-dominance and high polymorphism suit them better in the task of measuring genetic structure in H. armigera (Scott et al. 2003) than the RAPD markers used in the previous studies. The use of SSR markers for H. armigera was previously hampered by non-availability of the DNA sequence information. Recently, many SSR markers specific for H. armigera have been identified (Tan et al. 2001; Ji et al. 2003; Scott et al. 2004; Ji et al. 2005). Hence, the present preliminary study was conducted to evaluate genetic variability among H. armigera collected from six different host plants.

## Materials and Methods

### Helicoverpa armigera collection

**Figure 1:**
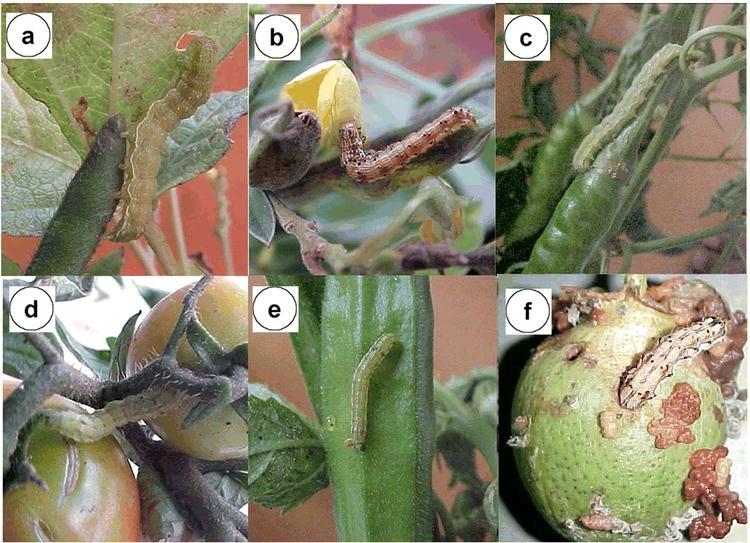
Occurrence of Helicoverpa armigera on different hosts, a. Blackgram, b. Redgram, c. Chili, d. Tomato, e. Bhendi, cf. Cotton

Collection of random samples of H. armigera was done during the month of September and October of 2003 on six different hosts including tomato, bhendi, blackgram, redgram, chili and cotton (Figure 1). About 50 larvae were collected for each host crop. The larval samples were collected at the rate of one larva per individual plant from 10 different plants selected at random within a field. Five different farmer’s fields were selected for the collection of the larvae. The larvae collected in the field were reared on the same host for three generations in the laboratory maintaining 100 individuals per generation. From the 100 laboratory reared insects in the second generation, one adult female per host plant was randomly selected for the isolation of genomic DNA and stored at −70° C.

### DNA extraction

The insects were washed thoroughly in double distilled water and the genomic DNA was prepared from the adult females using a modified CTAB method (Saghai Maroof et al. 1984). Briefly, the cleaned insects were ground with 1.0 ml of cetyl trimethyl ammonium bromide buffer (CTAB) 42%, 100 mM Tris-HCI (pH 8.0), 1.4 mM sodium chloride, 20 mM EDTA, 0.5 M glucose, 0.1% of 2-mercaptoethanol (added just prior to use) and suspended in the same buffer. The suspension was incubated at 65° C for 2 hours and then equal volume of chloroform: isoamylalcohol (24:1) was added. The suspension was centrifuged at 800 g for 15 minutes at 4° C. The upper aqueous layer was transferred to a fresh micro centrifuge tube taking care not to disturb the middle protein interface. DNA was precipitated by adding equal volume of ice-cold isopropanol. The precipitated DNA was spun at 8000 g and the resultant DNA pellet was washed with 70% ethanol and dissolved in 100 μl TE (Tris EDTA, 100 mM). Extracted DNA was further purified free of RNA contaminants by addition of 10 μ1/100 μl of RNase. The intact genomic DNA was visualized in a 0.8% agarose gel and quantified using a fluorometer (DyNa quant 200, Hoefer, www.hoeferinc.com/) following standard procedures. Depending upon the concentration, the DNA samples were diluted with sterile water to get a working solution of 20–25 ng/μl.

### PCR amplification

The genomic DNA from H. armigera females collected from six different hosts were subjected to polymerase chain reaction (PCR) using 10 different SSR primers (Tan et al. 2001;Ji et al. 2003) (Table 1) obtained from Sigma-Aldrich, (www.sigmaaldrich.com). PCR was carried out in 20 μl reaction mixture containing 50 ng DNA as the template. Genomic DNA 2.0 μ1 (25 ng), dNTPs 0.8 μ1 (2.5 mM), assay buffer 1 μl (10X), SSR forward primer 2 μ1 (20 μM), SSR reverse primer 2 μ1 (20 μM), Taq polymerase 0.15 μl (3 units), magnesium chloride 0.15 μl (25mM), sterile distilled water 3.7 μl, were added and PCR was performed in a DNA thermal cycler (MJ Research, Inc., www.mjr.com) programmed for 4 min at 94° C for initial denaturation. Following the initial denaturation the thermal cycler was programmed for 40 cycles of 1 min at 94° C for denaturation, 1 min at 52° C for annealing and 1 min at 72° C for extension. An additional cycle of 5 min at 72° C was also used for primer extension.

**Table 1:**
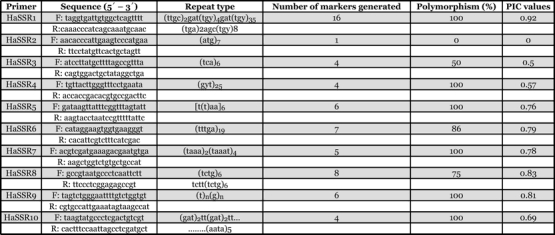
SSR markers utilized in the study, no. of markers generated for populations collected from different host and PIC values

### Electrophoresis of PCR products

PCR products were analyzed by electrophoresis in 6 per cent polyacrylamide gel electrophoresis run at 100 V/cm for 1.5 h in 0.5x TBE buffer. The banding pattern was visualized using the silver staining method (Panaud et al. 1996). The silver stained gel plates were allowed to dry and photographed. The molecular size of the amplified products was estimated using a 100 bp DNA marker (Fermentas Inc., www.fermentas.com). The samples were analyzed twice for all 10 primers to test the reproducibility of bands.

### Scoring of bands and statistical analysis

Based on log molecular weight of the co-migrating 100 bp DNA marker (Fermentas Inc., USA) and their migration distances scatter plots were established and trend lines with best fit was fitted. Based on the mathematical expression of the trend lines the molecular weight of the fragment corresponding to their migration distances was calculated. The individual DNA bands were scored as present or absent (1/0) in the amplification profile of each sample. Only clear bands with good resolution were scored. The scored marker data matrix was analyzed using the standard procedure in NTsys Pc-2.0 package (Rohlf 1998). The genetic distance or similarity was determined using the Dice coefficient (Dice 1945). The percentage of polymorphism was calculated as the proportion of the polymorphic markers to the total number of markers. The polymorphism information content value was also determined (Smith et al. 1997). A dendrogram was constructed after cluster analysis of the similarity coefficients by the un-weighted pair-group method analysis, UPGMA (Sneath and Sokal 1973) using NTsys Pc-2.0.

## Results and Discussion

**Figure 2:**
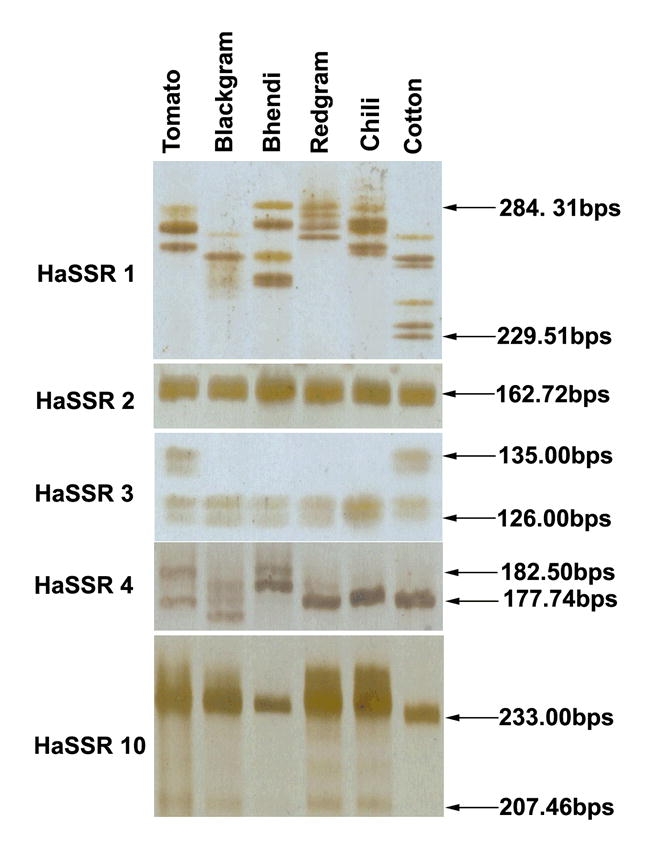
Simple sequence repeat (SSR) fragments generated from Helicoverpa armigera obtained from different host plants

The genetic variability of six populations of H. armigera collected from different host species (Fig. 1) was investigated by PCR analysis of DNA from one adult female randomly selected from each of these populations using 10 SSR primers. All ten primers listed produced scorable markers in each DNA sample and the primer HaSSR2 was found to produce a single monomorphic band for all DNA samples. Sample gels resulting from the HaSSR1, 2, 3, 4 and 10 primers across the populations collected from different hosts are presented in Figure 2. A total of 61 markers from 10 primers were available for analysis across the different populations. The highest numbers of 16 markers were produced by the primer HaSSR 1, followed by 8 markers by HaSSR 8 with high degree of polymorphism 75–100%. The primer HaSSR 1 was found to be highly informative to differentiate the host associated populations with a polymorphism information content value of 0.92 (Table1). The calculation of the dice coefficient values were based on the presence or absence of SSR bands. The coefficient values ranged from 0.348–0.741 (Table 2). The H. armigera populations occurring on tomato and bhendi were found to be closely related with a coefficient of 0.741, while the population occurring on cotton and blackgram was found to differ widely with a coefficient value of 0.348. The population on cotton was found to be distantly related to the others with lower dice coefficients.

**Table 2:**
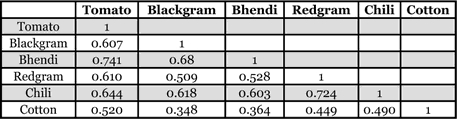
Dice coefficient matrix for Helicoverpa armigera populations collected from different host species using SSR markers

**Figure 3:**
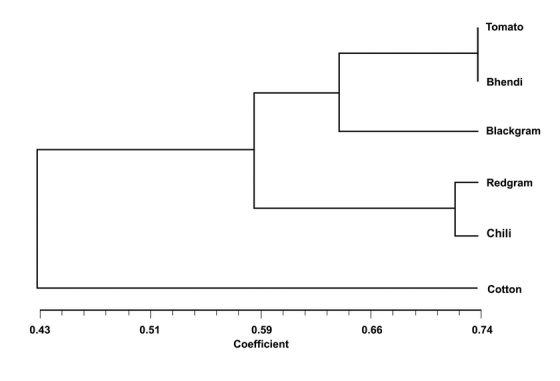
Dendrogram deduced from matrix of pairwise distances in SSR analysis between six populations of Helicoverpa armigera using the un-weighted pair-group method analysis, UPGMA.

The dice coefficient values were then utilized to cluster the data using the un-weighted pair-group method analysis of Sneath and Sokal (1973). The dendrogram (Fig. 3) revealed the existence of three principle clusters and a single sub-cluster. The population occurring on cotton stood out in a single cluster (A), while the population occurring on redgram and chili grouped together in cluster B, and the populations occurring on blackgram, bhendi and tomato grouped together in cluster C. The population occurring on tomato and bhendi were found together in a single sub-cluster C1. The bollworm, H. armigera inflicts severe damage on cotton worldwide. However, laboratory studies on the relative host preferences of H. armigera for cotton revealed that cotton was the host of lowest relative preference. Host preference hierarchies in H. armigera have been found to have a strong genetic component (Firempong and Zalucki 1990; Jallow and Zalucki 1996). However in areas of intense cotton cultivation a very high percentage of local bolloworm populations may feed exclusively on cotton at certain times of the growing season (Gould 1998). Experimental evidence has revealed that a previous experience with a host species increases the attractiveness to it due to host associated learning in H. armigera (Cunningham et al. 1998a, b; Cunningham et al. 1999). In the present study the grouping of the H. armigera populations indicated high similarity among populations collected from vegetable crops, while the population collected from the cotton crop was found to be more variable. This phenomenon indicates a strong genetic variability among H. armigera populations collected from different host plants. Moreover the earlier studies on the genetic variations of geographically isolated populations of H. armigera in India (Fakrudin et al. 2004) explained to some extent the susceptibility variation among such populations to insecticides (Armes et al. 1996) and to microbial pesticides such as Bacillus thuringiensis (Gujar et al. 2000). Shravankumar and Jagdishwar Reddy (2004) found differences in susceptibility to different insecticides among H. armigera populations collected from three hosts; chickpea, tomato and grapes. The authors suggested that this difference might be due to the variation in plant factors. The results of the present study also suggest that genetic variation among populations collected from different host plants might be due to host characteristics. Host-associated genetic differentiation has already been documented in moth families such as the Noctuidae (Pashley 1986), Tortricidae (Emelianov et al. 1995) and Prodoxidae (Groman and Pellmyr 2000).

The results of the present study also supports the view that polyphagous insects tend to be monophagic at the microecological level (Cunningham et al. 1999;Karowe 1989) as indicated by the genetic diversity between H. armigera populations collected from different host crops. Scott et al. 2003 found genetic shifts in H. armigera collections over monthly intervals and collection in any month was genetically distinct from all previous monthly collection. The author suggested that this might be due to the migration of populations from different locations. Ravi et al. (2005) examined the relative abundance of H. armigera on different host crops within a crop mosaic and found that the egg and larval numbers were higher in chickpea, tomato, sunflower and chili than on cotton and inferred that in a multicrop situation as occurs in India the other host crops listed above might act as an important natural refuge in central and southern India. The results of our study gains importance in a multicrop ecosystem as in India where a polyphagous insect has many of its hosts in the vicinity which may lead to interbreeding between isolated populations. Such an interbreeding phenomenon between varying host associated populations indicates the presence of natural refugia in multicrop environments as in India. However the degree of polyphagy expressed by individual H. armigera in the field is still unclear. Egg laying females could utilize a number of hosts or restrict laying to a single host (Cunningham et al. 1998a, b). Detailed field level investigations on the polyphagy of individual H. armigera and the mating behavior of such individual populations combined with evaluation of their genetic diversity remains to be done.
